# ZINC-22—A Free Multi-Billion-Scale Database of Tangible Compounds for Ligand Discovery

**DOI:** 10.1021/acs.jcim.2c01253

**Published:** 2023-02-15

**Authors:** Benjamin I. Tingle, Khanh G. Tang, Mar Castanon, John J. Gutierrez, Munkhzul Khurelbaatar, Chinzorig Dandarchuluun, Yurii S. Moroz, John J. Irwin

**Affiliations:** † Department of Pharmaceutical Chemistry, University of California San Francisco, 1700 4th St, Mailcode 2550, San Francisco, California 94158-2330, United States; ‡ Taras Shevchenko National University of Kyïv, 60 Volodymyrska Street, Kyïv 01601, Ukraine; § Chemspace LLC, 85 Chervonotkatska Street, Kyïv 02094, Ukraine

## Abstract

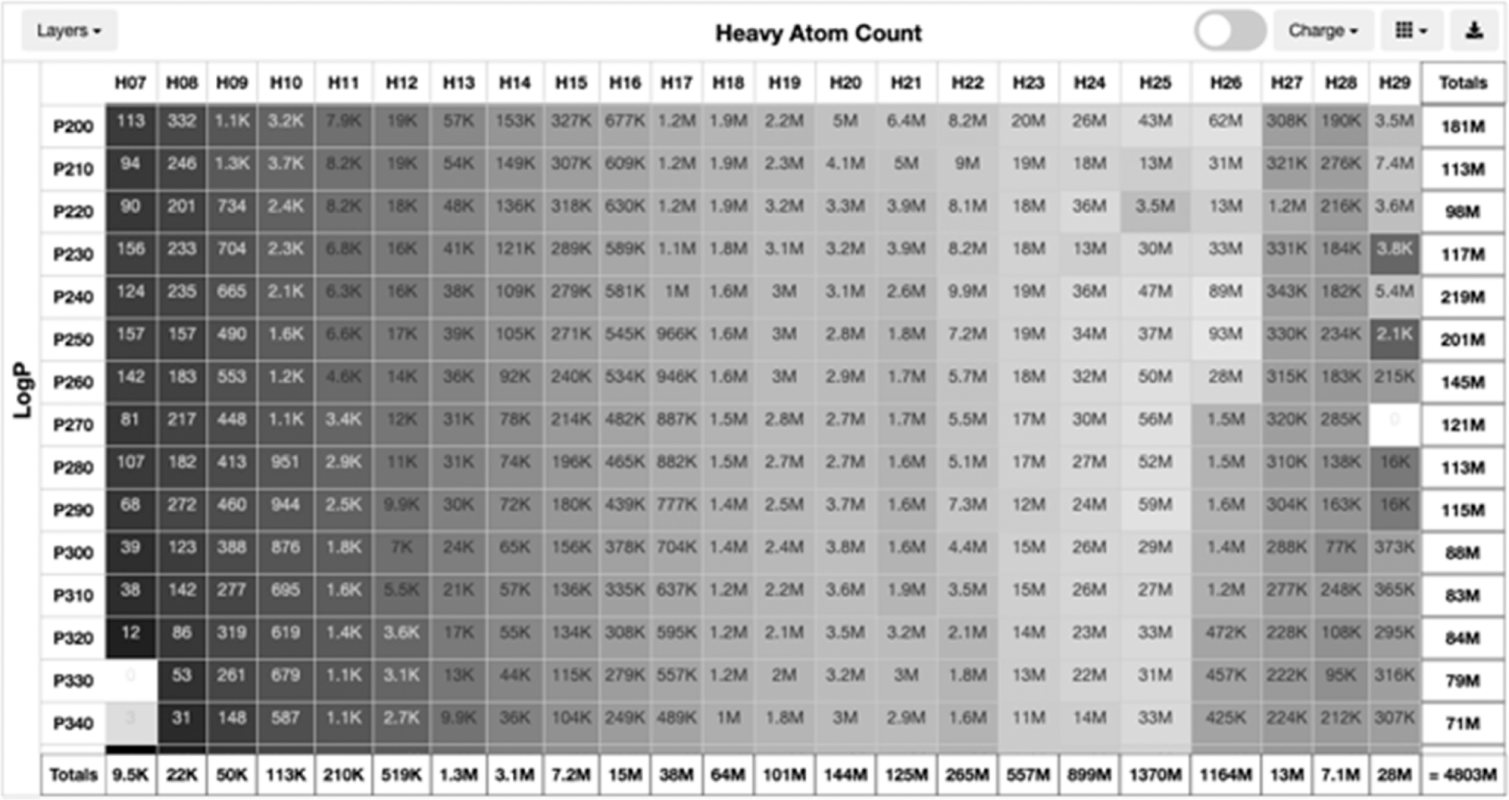

Purchasable chemical space has grown rapidly into the tens of billions of molecules, providing unprecedented opportunities for ligand discovery but straining the tools that might exploit these molecules at scale. We have therefore developed ZINC-22, a database of commercially accessible small molecules derived from multi-billion-scale make-on-demand libraries. The new database and tools enable analog searching in this vast new space via a facile GUI, CartBlanche, drawing on similarity methods that scale sublinearly in the number of molecules. The new library also uses data organization methods, enabling rapid lookup of molecules and their physical properties, including conformations, partial atomic charges, *c* Log *P* values, and solvation energies, all crucial for molecule docking, which had become slow with older database organizations in previous versions of ZINC. As the libraries have continued to grow, we have been interested in finding whether molecular diversity has suffered, for instance, because certain scaffolds have come to dominate via easy analoging. This has not occurred thus far, and chemical diversity continues to grow with database size, with a log increase in Bemis–Murcko scaffolds for every two-log unit increase in database size. Most new scaffolds come from compounds with the highest heavy atom count. Finally, we consider the implications for databases like ZINC as the libraries grow toward and beyond the trillion-molecule range. ZINC is freely available to everyone and may be accessed at cartblanche22.docking.org, via Globus, and in the Amazon AWS and Oracle OCI clouds.

## Introduction

The growth of readily available, make-on-demand (“tangible”) molecules creates new opportunities for ligand discovery.^2−9^ Tens of billions of new molecules, previously only accessible via more expensive bespoke synthesis, may now simply be ordered from a catalog. An important problem is how to screen these large libraries efficiently. Even apparently simple tasks, like searching for analogs, which are rapid for million-scale chemical libraries, are ill-suited to handle multi-10-billion scale collections because the indexes are too big to fit in rapid access computer memory. The problem is just as apparent for more complex questions like molecular docking, which, though they scale linearly, are computationally intensive. Computer clusters capable of docking hundreds of millions of molecules in days or weeks now struggle with billions. New methods to work with this space are urgently needed.

We wanted to revise the widely used ZINC platform^9−12^ to address key problems that medicinal chemists, structural biologists, and chemical biologists will inevitably encounter to engage with this growing tangible space. **First**, we wanted to address the apparently simple problem of seeking analogs for a particular compound or series of compounds. This was trivially done for libraries of several million molecules but collapses as one approached a billion molecules. A method to do this was described in a previous study;^9^ here, we focus on the development of a facile and integrated GUI, CartBlanche, to facilitate these searches and organize the results. **Second**, we continue to support molecular structure and property calculation for physical modeling, often molecular docking—an initial motivation for the database.^10^ As we move into the multi-billion molecule space, the organization of the data in ZINC has had to change. In building ZINC-22, we investigated data organization schemes that address challenges in disk access, rapid lookup, database distribution and download, and the relational structure of the database. **Third**, and perhaps most interesting from a chemical space and chemical information standpoint, we consider how the growth of the libraries has changed the properties of the molecules represented, their diversity, and what the limits to growth of a library like ZINC might be. **Finally**, as daunting and as exciting as our new world of 10–100 billion molecules is, it remains a tiny fraction of drug-like chemical space. To address this space, researchers are increasingly turning to active learning, artificial intelligence, and machine learning (AI/ML) techniques as well as unenumerated chemical space methods to improve the efficiency of drug discovery using large chemical libraries.^13−18^ In active learning, a model is first trained on a small set of labeled data and then used to select the most informative samples from the data set for labeling, allowing ultralarge libraries to be mined more efficiently. In this version of ZINC, we aspire to cater to this enthusiastic and growing community alongside our longstanding commitment to structure-based screening and cheminformatics.

In addressing these questions, we hope we have built a library and database that will support chemists, medicinal chemists, and chemical biologists as they begin to interrogate the vast new tangible chemical space that has emerged in the last four years. The strategies we adopt may also be useful to others as they build related tools to address this space.

## Results

A new database, ZINC-22, is freely available for access and download by everyone. The database contains over 37 billion enumerated, searchable, commercially available compounds in 2D, over 4.5 billion of which have been built in biologically relevant ready-to-dock 3D formats. The database can be searched online using whole-molecule similarity, substructure, and patterns in 2D. The database includes molecules up to 29 heavy atoms (HAC29) and can easily be extended to HAC34 or higher. Over 95% of available molecules up to HAC24 have been built in 3D, and over 80% up to HAC25. Most paragraphs in the Results section are supported with additional information in the Methods section and the Supporting Information. We take up the features of the new database in turn.

### Contents

ZINC-22 focuses on large libraries of make-on-demand compounds. It includes catalogs from Enamine (REAL), WuXi (GalaXi), and Mcule (Ultimate). Because in-stock compounds are important as an “informer set” that is often screened before a large-scale screen, ZINC-22 incorporates the ZINC20 informer set. The previous version of ZINC, ZINC20, continues to be maintained and contains all in-stock purchasable compounds, building blocks, and annotated compounds. From among the make-on-demand databases, ZINC20 only contains molecules that we had loaded prior to creating ZINC-22. (see the Methods section and Supporting Information S1)

#### The CartBlanche Web Interface

**Figure 1:**
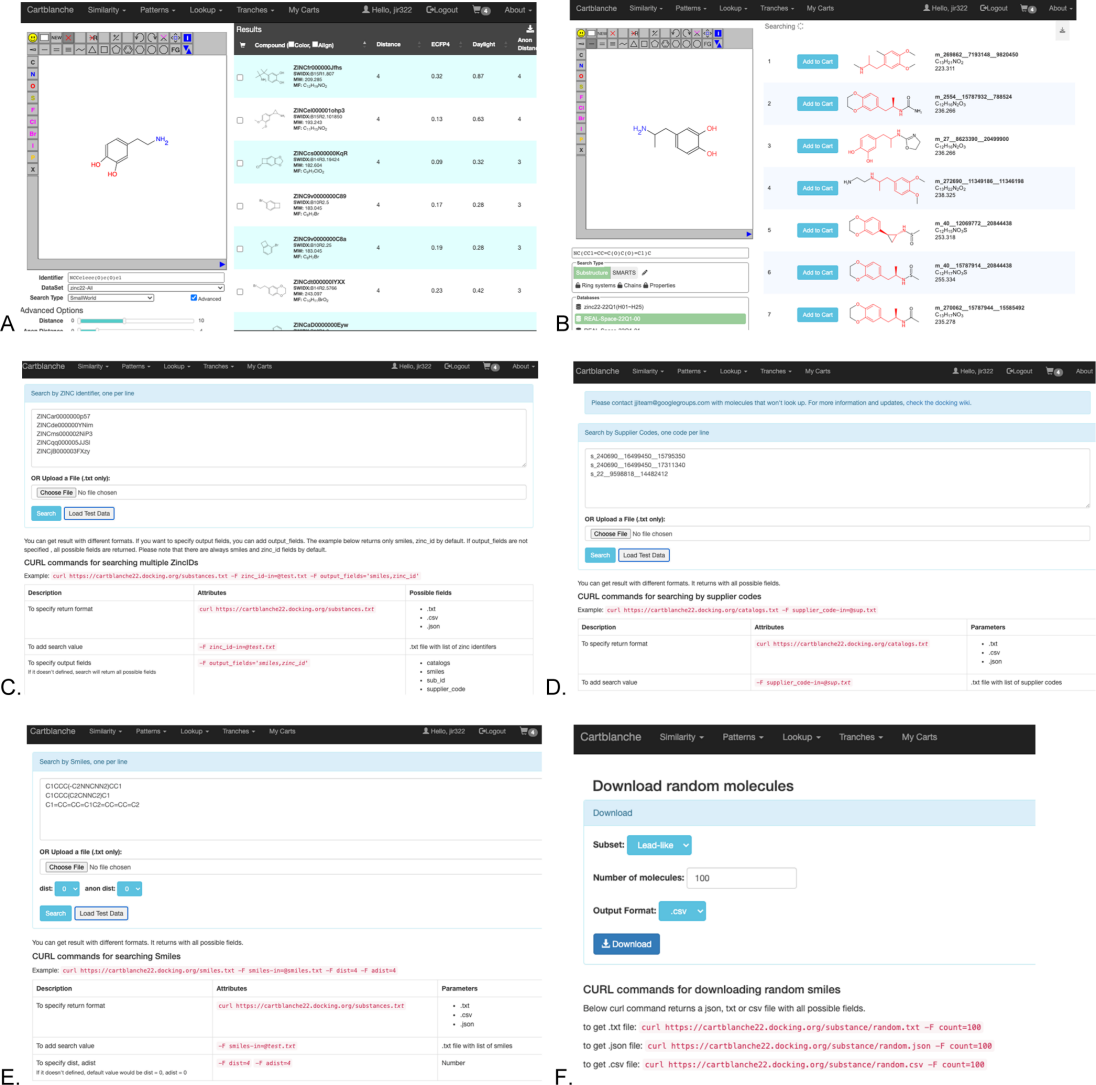
Cartblanche22.docking.org graphical user interface.^1^ (A) Molecular similarity and substructure search using SmallWorld. (B) Substructure and pattern search using Arthor. (C) Lookup by ZINC ID. (D) Lookup by supplier code. (E) Lookup by SMILES in bulk. (F) Random molecule selector.

We created a website, cartblanche22.docking.org, to access ZINC-22 (Figure 1). The interface has the following features: (1) molecular similarity search; (2) substructure search; (3) pattern search; (4) lookup by ZINC ID; (5) lookup by supplier code; (6) lookup multiple SMILES; (7) database subset selection and download; (8) random molecule selection; and (9) a shopping cart metaphor for organizing, curating, and preparing sets of molecules for purchase. Most features are available via the command line using curl or wget (see the Methods section) as well as via a web interface (Figure 1), which documents the command line use at the bottom of each page. Commercially available molecules that are disclosed on the Internet may not be patented, we are advised, and thus some database subsets, including ZINC-22, require that the user logs in. To further prevent public access, a second level of authentication is required to access private databases (see Supporting Information S0). We take up each feature in sequence.

### Molecular Similarity Search

This is also known as ABC—Analog By Catalog. The interface allows molecules similar to a given structure to be identified rapidly using SmallWorld,^19^ a tool developed by NextMove Software (Figure 1A), as used in ZINC20, improved.^9^ SmallWorld uses graph edit distance to perform the search and calculates two fingerprints for comparison with other methods and resorting. Molecules found may be loaded into the shopping cart for retention and prioritization (see below). The advanced graph-edit-distance options in the interface allow targeting of specific kinds of analogs, such as substructure, scaffold, and other types. For finding many compounds and their close analogs at once, a bulk search tool is available as a separate feature (see below). To protect the patentability of chemical space, we run multiple SmallWorld servers (see the Methodssection).

### Molecular Substructure and Pattern Search

ZINC-22 allows molecules containing a given substructure or molecular pattern expressed as SMILES or SMARTS to be found rapidly using Arthor (Figure 1B). The interface allows up to 20,000 molecules containing the substructure or pattern to be found and displayed interactively, often in seconds. Molecules that are found may be put in the shopping cart or downloaded using the menu in the top right. Up to 100,000 molecules may be downloaded using the download menu. If more than 100,000 molecules are desired, the user should use the Arthor tool in the TLDR interface (tldr.docking.org). To protect the patentability of chemical space, we ran multiple Arthor servers (see the Methodssection). SmallWorld (above) provides an alternative and faster approach to substructure search (see the Methods section).

### Lookup by ZINC Code

ZINC codes may be looked up one at a time or up to 1000 at once to find SMILES and purchasing information (Figure 1**C**). Following a screening campaign, the user may return here to look up the latest purchasing information for the codes and to put the available molecules and perhaps their close analogs into the shopping cart for purchasing. The interface supports both ZINC-22 codes as well as ZINC20 (ZINC15) codes. Lookup by ZINC code uses the Sn databases and ZINC20 (see the Methods section and Supporting Information). To use lookup by ZINC code, the user browses to cartblanche22.docking.org and then selects Lookup > by ZINC code from the popup menu. It is also possible to download the entire mapping of vendor codes to ZINC codes (see the Methods section), but as this information changes frequently, we recommend returning to the website to look up the latest purchasing information.

### Lookup by Supplier Code

Vendor purchasing codes can be looked up one at a time or up to 1000 at a time to find SMILES and ZINC ID rapidly (Figure 1D). This is a useful tool to ask whether particular molecules from a particular vendor are included in ZINC-22. Molecules that are found may be loaded into the shopping cart. Lookup by supplier codes uses the Sb database (see the Methods section and Supporting Information). To use lookup by supplier code, the user browses to cartblanche22.docking.org and then selects Lookup > by Supplier code from the pull-down menu.

### Lookup up Multiple SMILES

Molecules and their nearest neighbors may be looked up using this tool. Whereas the SmallWorld interface (above) allows molecules and their analogs to be found for one molecule at a time, here we allow up to 1000 SMILES to be searched against the ZINC-22 database in a single operation. The user may pick the degree of the match using the distance (dist) and anonymous distance (adist) parameters. Thus, dist = 0 is an exact match, adist = 0, dist = 1 allows for a single change (e.g., atom type, bond order) without a change in the molecular topology. The selection of adist = 1 allows for a single change in the topology (ring size increase or decrease, opening or closing a ring, addition or deletion of a single atom). For more details on distance and anonymous distance, please refer to the documentation for SmallWorld, which may be obtained free of charge by writing to NextMove Software. Molecules that are found may be loaded into the shopping cart. Lookup by SMILES uses SmallWorld indexes. To use lookup in bulk by SMILES, the user browses to cartblanche22.docking.org and selects Lookup > by SMILES from the popup menu. For more extensive and intensive searching, we recommend downloading the entire database in 2D (see Tranche Browser below) and performing the searches on the local computer, e.g., using RDKit or other tools.

### Database Subset Selection Using the Tranche Browser

**Figure 2:**
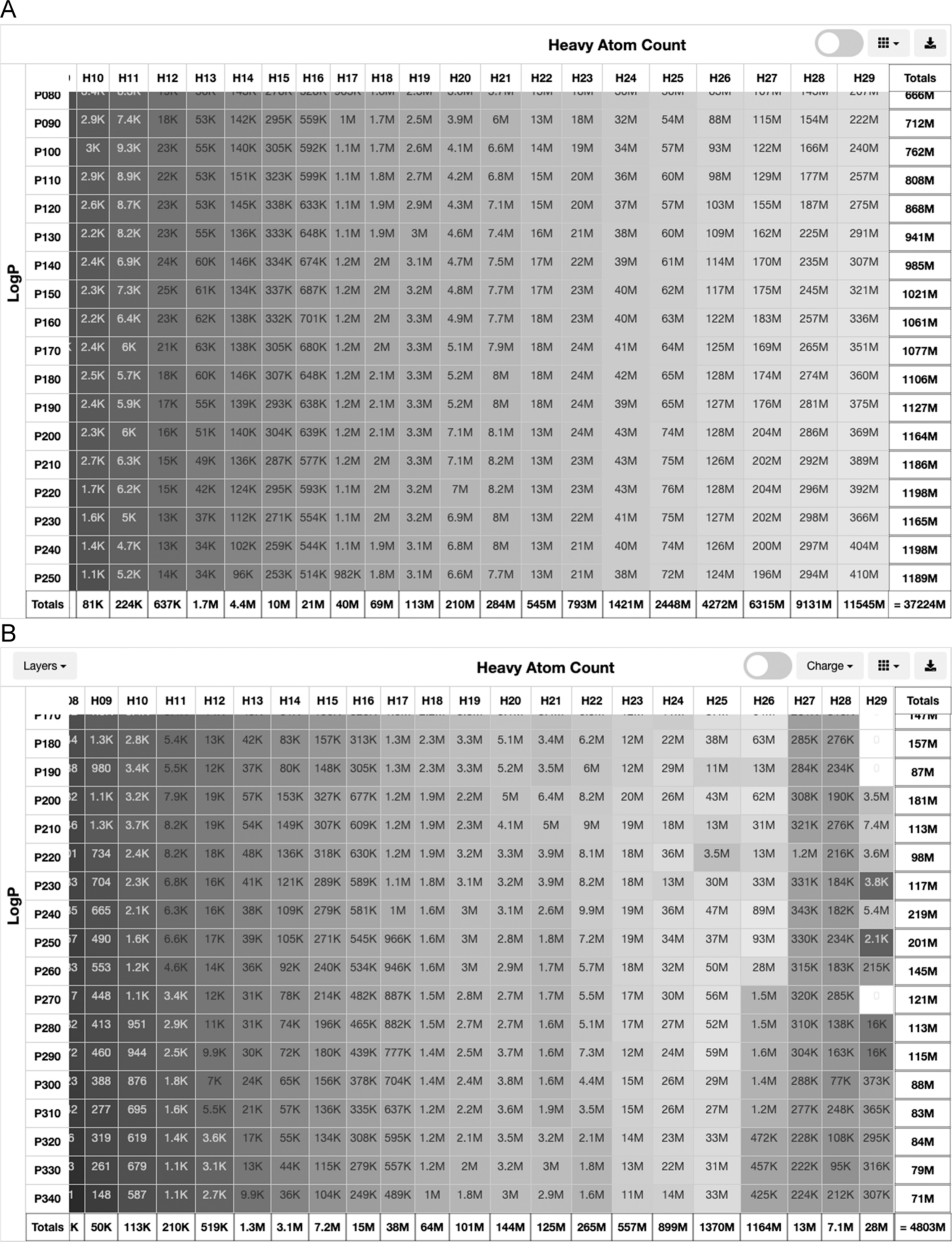
Database subset selection and download using the tranche browser. (A) 2D tranche browser. (B) 3D tranche browser.

The CartBlanche interface allows physical chemical space to be explored and prioritized. The user browses cartblanche22.docking.org and selects Tranches and then 2D. A graphical browser appears (Figure 2A), allowing chemical space to be selected along two axes: heavy atom count (horizontal) and lipophilicity (vertical). Common subsets such as “lead-like” and “fragment-like” may be selected using the selection pull-down menu (top right). Each square has an estimate of the number of molecules available in that tranche. Individual tranches may be toggled on and off by clicking on them, and entire rows and columns may be toggled by clicking on the row and column headings. When the selection is complete, a script may be downloaded to download or access the selected molecules in various formats.

### Random Molecules

CartBlanche has a random molecule set generator that can select a desired number of molecules from ZINC-22 at random. Using the available tranche data (how many molecules are in each tranche and which tranches are found in each database instance), a numerical distribution is generated. Using that distribution, each Sn database is assigned weights that are used to pull molecules randomly. After the distribution is generated, a database is picked, and a random molecule is retrieved. This process is repeated for the desired number of molecules. The random generator can also pull random molecules within a subset. In this case, distribution is generated for that subset (e.g., lead-like), and molecules will be picked from the respective tranches.

### Prioritize for Purchase Using the Shopping Cart

**Figure 3:**
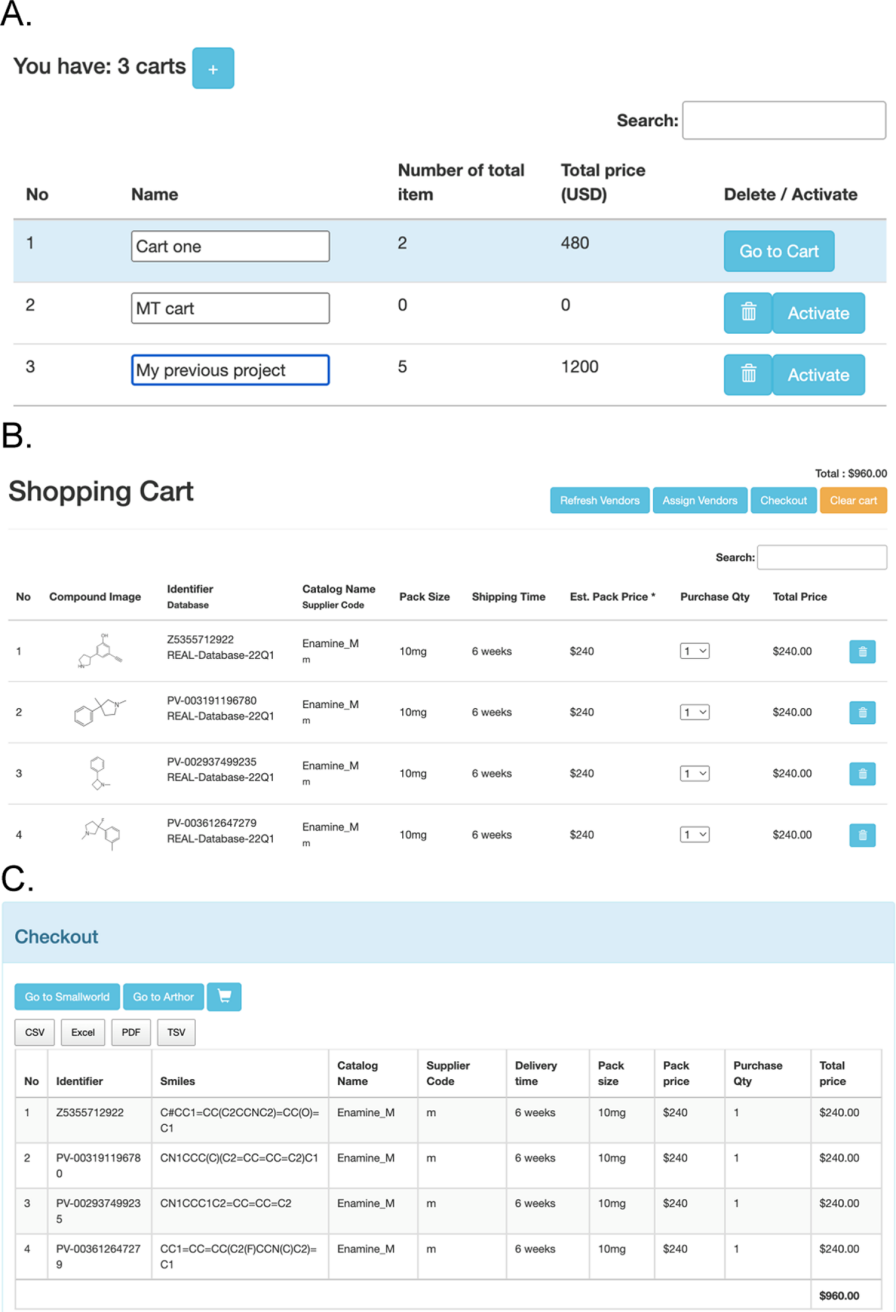
Shopping Carts to prioritize molecules in Cartblanche22.docking.org.^1^ (A) Manage multiple carts, only available to authenticated users. (B) Review and edit the cart. (C) Checkout reports to facilitate purchase. Prices are for rough guidance only.

CartBlanche contains a shopping cart feature that allows molecules found in a search to be retained for further processing (Figure 3). The cart supports reviewing molecules and their purchasing information, links to identify additional analogs, and removing individual molecules. An approximate price estimate is calculated in the hope that it will provide some useful guidance, but only the vendor can provide the true price. For users that register and sign in, multiple carts allow multiple projects to be pursued in parallel. The cart supports multiple formats, including Google Sheets, Excel, PDF, and plain text, to assist with routing this information to the vendor for a price quote.

#### Ready-to-Dock 3D Models

A second major area of development in the new version has been in the growth and reorganization of the way 3D models for docking are built, organized, and distributed. ZINC-22 is now a federation of many smaller databases (see the Methods section). By breaking the database up into smaller parts, each part can be prepared asynchronously, concurrently, and scalably (this was a problem in the earlier version of ZINC, and we suspect any similarly organized database because of the limitation of a single registration pipeline). The database files are organized in a four-dimensional “space”: heavy atom count, lipophilicity, charge, and format. An additional organizational dimension, layer, allows the database to be prepared in parallel and independently as separate layers, allowing scalability. Molecules are packaged into files of up to 5000 molecules each, intended for a docking calculation on a single CPU. ZINC is distributed through multiple servers, making it easier to access. ZINC is available in Amazon’s AWS cloud, in Oracle’s OCI cloud, as well as on our server at UCSF. From UCSF, ZINC can be accessed using curl, wget, Powershell, rsync, and Globus.

### Cartblanche22 for 3D

CartBlanche has a 3D tranche browser (Figure 2B) to allow the user to select areas of chemical space according to heavy atom count, calculated log *P*, and charge, as well as file format. The user can also select the download format. The user browses cartblanche22.docking.org and, from the menu, selects tranches and then either 2D or 3D. (Figure 3). We suggest using the selection tool (top right) to select lead-like or perhaps one of the other 10 predefined subsets and then fine-tuning the selection by clicking either on individual tranches or on row and column headings to toggle the selection. The user selects the desired charge from the charge selector (top center). The user clicks on the download button (top right) to select the download format and method. When the user clicks “download,” a script is downloaded. Some scripts are used to download directly to the computer where they are run (curl, wget, Powershell). Others are lists of files in the cloud which may be read directly by a docking program (AWS S3, Oracle OCI). In this case, no “download” is required. In 3D, four docking formats are available: mol2, sdf, pdbqt, and db2.

#### Characteristics of the Tangible Molecules

##### Number of Molecules in the Source Catalogs

ZINC-22 currently uses five source catalogs: Enamine REAL Database (5 B), Enamine REAL Space (29 B), WuXi (2.5 B), Mcule (128M), and ZINC20 in stock (4 M) (see the Methods section and Supporting Information S1). There are about 2.5 billion molecules up to HAC25 before stereochemical expansion and a further 21 billion between HAC26 and HAC30.

##### How Chemically Diverse is the Database?

**Figure 4:**
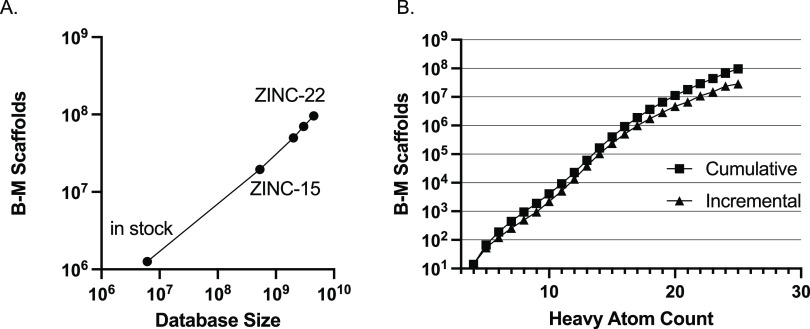
Bemis–Murcko (B-M) Scaffolds in ZINC. (A) Growth of unique scaffolds as the database has grown. (B) Cumulative number of scaffolds per heavy atom in ZINC-22.

Bemis–Murcko (B-M) scaffolds are calculated by removing terminal acyclic bonds and retaining a core ring structure, including any terminal exocyclic double-bonded atoms. B-M scaffolds are widely used^20−24^ as a pragmatic measure of structural diversity. We wondered how the number of B-M scaffolds increased with database size. We calculated the number of B-M scaffolds in the in-stock collection: 6,136,700 had 1,263,063 B-M scaffolds. For terminal ZINC15 and ZINC20 databases: 531,645,834 molecules had 19,590,914 B-M scaffolds. For ZINC-22 in October 2022: 4,500,000 billion molecules had 96,311,761 B-M scaffolds (Figure 4A).

We also wondered how the number of B-M scaffolds increased with the number of heavy atoms (Figure 4B). We plotted both the cumulative number of scaffolds with each additional heavy atom as well as the incremental number of scaffolds added with each additional atom. In the “fragment-like” range of 10–16 heavy atoms, each additional atom increases the number of B-M scaffolds by between 2- and 3-fold. In the small-lead-like range, this drops below 2-fold, and among the largest lead-like molecules (24–25 heavy atoms), it falls still further to about 1.2-fold growth in scaffolds with one additional atom. Although the trend in the ratio of new scaffolds with each additional atom is seen as generally declining, the exact trend remains unclear.

##### Ongoing Work

When we are running at full speed, we can add about 300 million new molecules to ZINC-22 every month. We update these in the AWS and OCI clouds about once a week and tweet about ZINC-22 growth @chem4biology.

##### Limits to Growth

Some groups have begun to abandon full database enumeration, preferring instead to work in building block space.^6,25−29^ Our group, with its commitment to our approach to molecular docking, currently requires enumerated databases. Fortunately, software such as Arthor and SmallWorld has allowed us to continue to index and offer rapid public search within multi-10-billion databases. We think our new modular design of ZINC-22 will allow us to grow the database by another log unit or two, above a trillion molecules but perhaps not ten trillion. This cannot go on forever. We acknowledge the logic of avoiding full database enumeration using unenumerated combinatorial spaces.^27^ Still, we believe there are reasons to continue full database enumeration as long as we are able. Work from our lab suggests that as the database grows, docking can identify ever-better-fitting molecules.^30^

## Discussion

Three themes emerge from this work. **First**, a new database, ZINC-22, is now freely available on our website. Improved tools for interrogating this database are now available, including whole-molecule similarity, substructure, and pattern search. **Second**, the 3D database has been reorganized to make it more scalable. The database is now distributed on two cloud platforms for ease of access. **Third**, the database is chemically and structurally diverse, reflecting the enormous effort by vendors to add new reaction schemes and, particularly, new building blocks. We take up each of these points in turn.

A new database is freely available. In 2D, the database can be consulted in several ways. SmallWorld allows rapid graph-edit-distance search of chemical space using precalculated anonymous graphs, while Arthor allows both substructure and pattern searches. Both tools may be accessed directly or via a shopping cart metaphor called cartblanche22.docking.org, allowing the results of searches to be curated prior to ordering. CartBlanche22 is freely available.

In 3D, the database is organized in tranches of up to 5000 molecules each, organized by heavy atom count (HAC), lipophilicity (calculated log *P*), net molecular charge, and file format. This organization has the advantage that it allows the selection of molecules by these four features, and the molecules are grouped in work units that correspond to perhaps an hour of docking. For instance, it is easy to dock lead-like cations with 22 heavy atoms in db2 flexibase format because the molecules are prepackaged this way. The database is hosted by two independent cloud providers, AWS and OCI, as well as from our improved website, making the database easier to download. A tranche browser simplifies subsetting the database, allowing the database screened to be tailored to the aims of each project. One billion molecules require about 1 TB for each of mol2, sdf, and pdbqt formats, while for db2 flexibase format, the space required is around 200 TB (see Supporting Information S9).

ZINC-22 is designed to scale in several ways. First, it allows tranches of chemical space to be built independently and asynchronously by colleagues who wish to contribute. This is achieved by compartmentalizing physical chemical space by heavy atom count and calculated log *P*. Second, the underlying design is a federation of databases, allowing it to be sharded across hundreds of computers; for instance, as of writing ZINC-22 is composed of 174 independent Postgres 12.0 databases, 110 Sn, and 64 Sb (see the Methods section), and this can be easily scaled as the database grows. Third, the number of files used to store and distribute ZINC-22 has been reduced by packaging up to 5000 physically similar molecules together, reducing the strain on the file system. Using our current hardware, we can build around 11 M molecules per day, each with hundreds of precalculated conformations, partial atomic charges, and solvation energies suitable for docking on a sustained basis. At this pace, and with this structure, we expect the dockable subset of ZINC-22 to grow to over 8 billion molecules by the end of 2023.

As the library has grown, it is possible to imagine it becoming dominated by a small number of scaffolds that support facile elaboration, reducing diversity. This, however, has not been the case. As the library has added molecules, growing from hundreds of millions in ZINC15 to the current 4.5 billion, the number of Bemis–Murcko scaffolds has grown correspondingly and linearly with the overall database size. Over the past 5 years, for every 10 molecules added to the library, one new scaffold has been added (Figure 4a). Most of the scaffold diversity comes with the highest heavy atom count. Just the molecules in HAC24 and HAC25 contribute about twice as many scaffolds as all of HAC06-HAC23. The growth in library diversity has supported the ability to continue to discover novel scaffolds for targets and the steady improvement in docking scores and affinities.^29−31^

The database does retain important limitations. The db2 flexibase files used for docking with DOCK3.8 and containing precalculated conformations are large and slow to transfer. Four and a half billion molecules in db2 flexibase format consume nearly a petabyte and would require nearly three months to download at 1 Gbps. Interactive substructure and pattern searches with Arthor are currently limited to 20,000 molecules each for interactive use. Unlike previous versions of ZINC, individual molecules cannot be downloaded in 3D, though they may be recalculated through the newbuild3d tool of tldr.docking.org. Because of the way it is packaged into 5000 molecule tranches, updates, including the removal of no-longer-available molecules, are more difficult.

These concerns should not obscure the important advantages of ZINC-22. New tools provide 2D search capabilities wrapped in a shopping cart metaphor. The database is hosted by two independent cloud providers, ready for use, as well as being downloadable from our website. The database can be easily subsetted and is highly diverse. It is built to scale and should nearly double in size in the coming year to 8 billion molecules. As ever, it is openly available to the community without restriction at https://cartblanche22.docking.org.

## Methods

### Software Versions

We used RDKit version 2020_03, OpenBabel version 2.3.2, and Molinspiration version 2018. We used Postgres 12.2 for the database to host ZINC and Python 3.6.8 or later and CentOS7 operating systems. We used Flask v1.1.2, Celery v5.2.0, and Redis v6.0.9.

### Catalogs

The ZINC-22 database is built from four source catalogs. Enamine, WuXi, and Mcule catalogs were sourced directly from the vendor websites (see Supporting Information S1). ZINC20 continues to be maintained, and the in-stock portion is incorporated into ZINC-22 as layer “g.” ZINC20 remains our best attempt at keeping track of the over 200 smaller catalogs, while ZINC-22 focuses on large catalogs that are too big for ZINC20.

### Overall Database Structure and Sharding

We allocate molecules into bins or tranches in physical chemical space. We calculate the heavy atom count and the log *P* using RDKit. We then compute the bin based on our script (see Supporting Information S2).

### Database Numbering

We number molecules as follows. ZINC IDs are 16 characters: “ZINC” followed by two tranche characters, followed by two database source parameters (“00” for commercial libraries in ZINC-22), followed by a monotonically increasing number in an 8-character field. Numbers are radix 62 selected from the alphabet 0–9a-zA-Z. The two tranche characters are heavy atom count, and the second is for log *P* (see Supporting Information S3). For backward compatible treatment of ZINC20 (and ZINC15, ZINC12, etc.) codes, “00” for HAC and Log *P* indexes indicates this molecule is from one of these older databases.

### Computing Infrastructure

We do all curation and host all public websites and services of ZINC-22 on our cluster of about 1700 cores. (see Supporting Information S4).

### Overall Database Structure and Sharding

Each tranche may be processed independently of any other tranche, allowing for remote and asynchronous database building. The purchasing information and SMILES are loaded into two types of Postgres database, each optimized for one task. One database type is dedicated to lookups by ZINC ID (internally called Sn, see Supporting Information S5), while the others are for lookup by supplier code (Sb, see Supporting Information S6). A “common database” serves as a repository for static data used by ZINC-22 systems (see Supporting Information S7). At this moment, there are 110 Sn database instances and 64 Sb database instances, divided over 14 computers (see Supporting Information S4). Sn and Sb databases are only accessible via CartBlanche and are not directly accessible to the public. The relational database structure allows for enforcement of a unique ID number for each SMILES, and each ZINC ID within each database, as well as maintaining referential integrity of purchasing information, including duplicates. This structure also helps ensure that each molecule is represented only once in the entire set of databases. Adding additional computers as well as Sn and Sb databases as needed is possible, allowing the database to scale—another two log units we hope—on commodity computer hardware.

### 2D Database Preparation

Source catalogs are processed and loaded into the databases (2D only) using the new publicly available scripts (see Supporting Information S8). SMILES are neutralized with MiTools (molinspiration.com), which also filters out most incorrectly coded molecules.

### 3D Database Building

We use ChemAxon’s JChem package and the command line tool CXCALC to calculate protonation states and tautomers at or near physiologically relevant pH. Each protomer is rendered into 3D using Corina (Molecular Networks GmbH) and conformationally sampled using Omega (OpenEye Scientific Software, Santa Fe NM). Atomic charges and desolvation penalties are calculated using AMSOL 7.1 and our previously published protocol.^32^ We perform a strain calculation to calculate the relative energies of conformations.^33^ Files are formatted for docking as flexibase files, mol2, sdf, and pdbqt.

### CartBlanche Interface

CartBlanche is a molecule shopping cart and ZINC-22 search tool used to retrieve and display molecule information. The application is built using the Python web framework Flask (v1.1.2). To avoid long front end wait times after submitting a request, the application uses a Celery (v5.2.0) task queue paired with Redis (v6.0.9) to run search processes in the background. ZINC-ID search can retrieve information for a list of molecules. When a list of ZINC IDs is passed in, the ids are processed to extract tranche information. Next, we prepare a map in which the database instance of each molecule should be found. This tranche-database map is then used to search the relevant Tin database instances for the Substance ID in the respective database. The result in the interface shows all available information about the molecule: SMILES, substance ID, tranche information, catalogs, and supplier codes.

Supplier code search uses a similar process to ZINC-ID search, with the extra step of having to gather a list of suppliers that can be found in specific databases. Antimony (Sb) is a database system that contains lists of which supplier ids can be found in each database. This initial step allows us to search only those databases where we expect to find matches. When a list of Supplier IDs is passed in, each ID is looked up in Antimony, and a map of TIN databases and supplier ids is returned. Once that map is collected, a search is run in each of those databases to find all molecules containing the relevant supplier code. Like the ZINC-ID search, this also pulls all available information about the molecule.

Searching for molecules by SMILES uses SmallWorld (v5.2) to perform a similarity search. A list of SMILES and two parameters (distance and anonymous distance) is passed to a SmallWorld Java executable that is called using a Python subprocess. The result contains a list of matching ZINC IDs with no other information. That list is then sent to the ZINC-ID search algorithm, which pulls the relevant data for each molecule.

### Multiple SmallWorld and Arthor Servers

To defend the patentability of chemical space, we password-protect some databases to prevent public disclosure. This required running multiple servers. Thus, the sw.docking.org and arthor.docking.org servers, called “Public” within CartBlanche, are public, previously disclosed molecules, with no password protection. Correspondingly, swp.docking.org and arthorp.docking.org, also called “Private” within CartBlanche, are only available using a password, to protect patentability (Supporting Information S0). Private databases are only visible inside CartBlanche when a user has logged in. Anonymous users only have access to Public databases.

### Upload to Cloud

We upload ZINC-22 to AWS using the AWS CLI weekly. We upload ZINC-22 to OCI using the OCI CLI weekly. We tweet about database updates @chem4biology.

### Policies in Effect

ZINC-22 would be much larger without policies to focus attention on the areas of chemical space we feel are most important. Thus, ZINC-22 aims to load comprehensively all commercially available, biologically relevant molecules up to HAC25. From H26 to H34, our policy is to load as we can, but we do not have the capabilities to be comprehensive. We favor molecules with log *P* less than four for solubility. Currently, no log *P* policy is enforced because the vendors generate only about 1% of molecules with log *P* over five and fewer than 7% between four and five, a rate we find acceptable.

### Asynchronous Arthor Search Using ZINC-22

Arthor searches are CPU- and memory-intensive and can take a long time to run, particularly for complex queries and/or ones that result in many molecules. To provide a reliable public service, the interactive use of Arthor is limited to 20,000 molecules in the result. Users who require more molecules may use the asynchronous Arthor search tool. To use this feature, the user signs into TLDR.docking.org. Using the Arthor tool, the user selects Substructure or Pattern and specifies the substructure or pattern. When complete, the user receives an email with download instructions.

### Difference between Substructure Search in SmallWorld and Arthor

Substructure search in SmallWorld and Arthor is almost identical, but there are important performance trade-offs and some limiting cases. SmallWorld scales better than Arthor when the database is bigger than RAM, likely for multi-billion-scale databases like ZINC. Arthor scales linearly with database size, so 5 billion molecules take 5 times as long as 1 billion to search, provided it all fits in RAM. SmallWorld search time is nonlinear: close neighbors are found almost instantly, but more distant matches take much longer. SmallWorld often cannot find very distant hits, for instance, benzene vs fullerene, whereas Arthor can. Arthor is good at finding PAINS and functional group patterns, SmallWorld is not.

### Download Purchasing Information

We recommend downloading purchasing information only for those molecules the user wishes to purchase. To do this, the user should use cartblanche22.docking.org. The user selects search by ZINC ID and specifies the codes and Search. This will find the molecules. The user may put the molecules into a cart and proceed to Checkout. We do not recommend it, but the user may download a copy of all purchasing information for every molecule in ZINC. To do this, the user goes to files.docking.org/zinc22/vendors_zincid_map/current/ where the files may be downloaded. We do not recommend doing this because the information will rapidly become stale. Using our website just prior to ordering is the best way to obtain current purchasing information.

### Programmatic Queries of ZINC-22

The following features are supported on the command line. First, files may simply be downloaded either from our website or the two cloud providers we currently support. We recommend the use of the tranche browser for making this selection, but the user may choose to use the wild-card features of wget, rsync, globus, and other tools to select and download files. Second, in CartBlanche22, each query page includes instructions for querying the database on the command line. Thus, at the bottom of the page cartblanche22.docking.org/search/byzincid/, the user will find the following general query structure, together with detailed instructions for each parameter. This command is also documented online at wiki.docking.org/index.php/Zinc22:Searching.

curl https://cartblanche22.docking.org/substances.txt-Fzinc_id-in=@test.txt-Foutput_fields=’smiles,zinc_id’

Likewise, for command line search by supplier code, the web interface cartblanche22.docking.org/search/bysupplier/ includes detailed documentation of the command line use of this feature.

curl https://cartblanche22.docking.org/catitems.txt-Fsupplier_code-in=@sup.txt

Likewise, for search by SMILES, which is accessed via the page cartblanche22.docking.org/search/bysmiles/, the command line use of this feature is described at the bottom of the page:

curl https://cartblanche22.docking.org/smiles.txt-Fsmiles-in=@smiles.txt-Fdist=4-Fadist=4

Finally, the feature allowing random selection of molecules from either the entire database or the lead-like subset, the page cartblanche22.docking.org/search/random/ documents the command line use of this feature:

curl https://cartblanche22.docking.org/substance/random.txt-Fcount=100

### Build Individual Molecules in 3D

Unlike its predecessors, ZINC-22 does not allow the download of individual molecules in 3D formats. However, we do support rebuilding molecules in 3D using our website. Use this tool as follows. The user browses TLDR.docking.org and signs in. The user selects newbuild3d and uploads the SMILES to be built. We accept a maximum of 50,000 molecules per transaction. The user will receive an email when the molecules are ready to download. Building typically takes 1 hour to build each 400 molecules, but this can vary considerably based on the system load.

## Data Availability

The software developed for and described in this paper is available in Github in the following repositories under https://github.com/docking-org/: zinc22-2d—the 2d curation and loading pipeline, cartblanche22—the CartBlanche22 website, TLDR—the TLDR server, tldr-modules—the TLDR individual modules, and zinc22-3d and zinc22-3d-build—the 3D building pipeline. All data produced for this project and described in this paper are freely available from our website at files.docking.org/zinc22/ and also on AWS and OCI via the respective open data programs. Some data is password-protected to protect future patentability of chemical matter (see **Supporting Information**, Files sizes in ZINC-22). The data in OCI and AWS clouds is freely available.

## Supporting Information Available

Access to databases to prevent molecules becoming unpatentable; Source catalog contributions to ZINC-22; Sharding script. ZINC-22 numbering; Software and Hardware overview; Sn system overview; Sb system overview; Common Database Schema Overview; Important management scripts for ZINC-22 (PDF)

## References

[ref2] Gorgulla C.; Boeszoermenyi A.; Wang Z. F.; Fischer P. D.; Coote P. W.; Padmanabha Das K. M.; Malets Y. S.; Radchenko D. S.; Moroz Y. S.; Scott D. A.; Fackeldey K.; Hoffmann M.; Iavniuk I.; Wagner G.; Arthanari H. An Open-Source Drug Discovery Platform Enables Ultra-Large Virtual Screens. Nature 2020, 580, 663–668. 10.1038/s41586-020-2117-z. 32152607 PMC8352709

[ref3] Lyu J. K.; Wang S.; Balius T. E.; Singh I.; Levit A.; Moroz Y. S.; O’Meara M. J.; Che T.; Algaa E.; Tolmachova K.; Tolmachev A. A.; Shoichet B. K.; Roth B. L.; Irwin J. J. Ultra-Large Library Docking for Discovering New Chemotypes. Nature 2019, 566, 224 10.1038/s41586-019-0917-9. 30728502 PMC6383769

[ref4] Grebner C.; Malmerberg E.; Shewmaker A.; Batista J.; Nicholls A.; Sadowski J. Virtual Screening in the Cloud: How Big Is Big Enough?. J. Chem. Inf. Model. 2020, 60, 4274–4282. 10.1021/acs.jcim.9b00779. 31682421

[ref5] Sunseri J.; Koes D. R. Virtual Screening with Gnina 1.0. Molecules 2021, 26, 7369 10.3390/molecules26237369. 34885952 PMC8659095

[ref6] Bellmann L.; Penner P.; Gastreich M.; Rarey M. Comparison of Combinatorial Fragment Spaces and Its Application to Ultralarge Make-on-Demand Compound Catalogs. J. Chem. Inf. Model. 2022, 62, 553–566. 10.1021/acs.jcim.1c01378. 35050621

[ref7] Klingler F. M.; Gastreich M.; Grygorenko O. O.; Savych O.; Borysko P.; Griniukova A.; Gubina K. E.; Lemmen C.; Moroz Y. S. Sar by Space: Enriching Hit Sets from the Chemical Space. Molecules 2019, 24, 3096 10.3390/molecules24173096. 31454992 PMC6749418

[ref8] Hoffmann T.; Gastreich M. The Next Level in Chemical Space Navigation: Going Far Beyond Enumerable Compound Libraries. Drug Discovery Today 2019, 24, 1148–1156. 10.1016/j.drudis.2019.02.013. 30851414

[ref9] Irwin J. J.; Tang K. G.; Young J.; Dandarchuluun C.; Wong B. R.; Khurelbaatar M.; Moroz Y. S.; Mayfield J.; Sayle R. A. Zinc20-a Free Ultralarge-Scale Chemical Database for Ligand Discovery. J. Chem. Inf. Model. 2020, 60, 6065–6073. 10.1021/acs.jcim.0c00675. 33118813 PMC8284596

[ref10] Irwin J. J.; Shoichet B. K. Zinc−a Free Database of Commercially Available Compounds for Virtual Screening. J. Chem. Inf. Model. 2005, 45, 177–182. 10.1021/ci049714+. 15667143 PMC1360656

[ref11] Irwin J. J.; Sterling T.; Mysinger M. M.; Bolstad E. S.; Coleman R. G. Zinc: A Free Tool to Discover Chemistry for Biology. J. Chem. Inf. Model. 2012, 52, 1757–1768. 10.1021/ci3001277. 22587354 PMC3402020

[ref12] Sterling T.; Irwin J. J. Zinc 15 - Ligand Discovery for Everyone. J. Chem. Inf. Model. 2015, 55, 2324–2337. 10.1021/acs.jcim.5b00559. 26479676 PMC4658288

[ref1] Tingle B.; Castanon J. Cartblanche22, https://cartblanche22.docking.org/.

[ref13] Schmidt R.; Klein R.; Rarey M. Maximum Common Substructure Searching in Combinatorial Make-on-Demand Compound Spaces. J. Chem. Inf. Model. 2022, 62, 2133–2150. 10.1021/acs.jcim.1c00640. 34478299

[ref14] Arul Murugan N.; Ruba Priya G.; Narahari Sastry G.; Markidis S. Artificial Intelligence in Virtual Screening: Models Versus Experiments. Drug Discovery Today 2022, 27, 1913–1923. 10.1016/j.drudis.2022.05.013. 35597513

[ref15] Woodward D. J.; Bradley A. R.; van Hoorn W. P. Coverage Score: A Model Agnostic Method to Efficiently Explore Chemical Space. J. Chem. Inf. Model. 2022, 62, 4391–4402. 10.1021/acs.jcim.2c00258. 35867814

[ref16] Khalak Y.; Tresadern G.; Hahn D. F.; de Groot B. L.; Gapsys V. Chemical Space Exploration with Active Learning and Alchemical Free Energies. J. Chem. Theory Comput. 2022, 18, 6259–6270. 10.1021/acs.jctc.2c00752. 36148968 PMC9558370

[ref17] Yang Y.; Yao K.; Repasky M. P.; Leswing K.; Abel R.; Shoichet B. K.; Jerome S. V. Efficient Exploration of Chemical Space with Docking and Deep Learning. J. Chem. Theory Comput. 2021, 17, 7106–7119. 10.1021/acs.jctc.1c00810. 34592101

[ref18] Bellmann L.; Penner P.; Rarey M. Topological Similarity Search in Large Combinatorial Fragment Spaces. J. Chem. Inf. Model. 2021, 61, 238–251. 10.1021/acs.jcim.0c00850. 33084338

[ref19] NextMove Software, I. Smallworld, https://www.nextmovesoftware.com/smallworld.html, 2022.

[ref20] Nitulescu G. M. Quantitative and Qualitative Analysis of the Anti-Proliferative Potential of the Pyrazole Scaffold in the Design of Anticancer Agents. Molecules 2022, 27, 3300 10.3390/molecules27103300. 35630776 PMC9146646

[ref21] Naveja J. J.; Vogt M. Automatic Identification of Analogue Series from Large Compound Data Sets: Methods and Applications. Molecules 2021, 26, 5291 10.3390/molecules26175291. 34500724 PMC8433811

[ref22] Zahoránszky-Kőhalmi G.; Sheils T.; Oprea T. I. Smartgraph: A Network Pharmacology Investigation Platform. J. Cheminform 2020, 12, 5 10.1186/s13321-020-0409-9. 33430980 PMC6974502

[ref23] Parks C.; Gaieb Z.; Amaro R. E. An Analysis of Proteochemometric and Conformal Prediction Machine Learning Protein-Ligand Binding Affinity Models. Front. Mol. Biosci. 2020, 7, 93 10.3389/fmolb.2020.00093. 32671093 PMC7328444

[ref24] Li Y.; Hu J.; Wang Y.; Zhou J.; Zhang L.; Liu Z. Deepscaffold: A Comprehensive Tool for Scaffold-Based De Novo Drug Discovery Using Deep Learning. J. Chem. Inf. Model. 2020, 60, 77–91. 10.1021/acs.jcim.9b00727. 31809029

[ref25] Peng Z. Very Large Virtual Compound Spaces: Construction, Storage and Utility in Drug Discovery. Drug Discovery Today: Technol. 2013, 10, e387–94. 10.1016/j.ddtec.2013.01.004. 24050135

[ref26] Bajorath J. Extending Accessible Chemical Space for the Identification of Novel Leads. Expert Opin. Drug Discovery 2016, 11, 825–829. 10.1080/17460441.2016.1210126. 27383145

[ref27] Warr W. A.; Nicklaus M. C.; Nicolaou C. A.; Rarey M. Exploration of Ultralarge Compound Collections for Drug Discovery. J. Chem. Inf. Model. 2022, 62, 2021–2034. 10.1021/acs.jcim.2c00224. 35421301

[ref28] Müller J.; Klein R.; Tarkhanova O.; Gryniukova A.; Borysko P.; Merkl S.; Ruf M.; Neumann A.; Gastreich M.; Moroz Y. S.; Klebe G.; Glinca S. Magnet for the Needle in Haystack: ″Crystal Structure First″ Fragment Hits Unlock Active Chemical Matter Using Targeted Exploration of Vast Chemical Spaces. J. Med. Chem. 2022, 65, 15663–15678. 10.1021/acs.jmedchem.2c00813. 36069712

[ref29] Alon A.; Lyu J.; Braz J. M.; Tummino T. A.; Craik V.; O’Meara M. J.; Webb C. M.; Radchenko D. S.; Moroz Y. S.; Huang X. P.; Liu Y.; Roth B. L.; Irwin J. J.; Basbaum A. I.; Shoichet B. K.; Kruse A. C. Structures of the Sigma2 Receptor Enable Docking for Bioactive Ligand Discovery. Nature 2021, 600, 759–764. 10.1038/s41586-021-04175-x. 34880501 PMC8867396

[ref30] Lyu J.; Irwin J. J.; Shoichet B. K. Modeling the Expansion of Virtual Screening Libraries ChemRxiv 2022, 2022, 10.26434/chemrxiv-2022-6lv34-v2. PMC10243288 36646956

[ref31] Lyu J.; Wang S.; Balius T. E.; Singh I.; Levit A.; Moroz Y. S.; O’Meara M. J.; Che T.; Algaa E.; Tolmachova K.; Tolmachev A. A.; Shoichet B. K.; Roth B. L.; Irwin J. J. Ultra-Large Library Docking for Discovering New Chemotypes. Nature 2019, 566, 224–229. 10.1038/s41586-019-0917-9. 30728502 PMC6383769

[ref32] Wei B. Q.; Baase W. A.; Weaver L. H.; Matthews B. W.; Shoichet B. K. A Model Binding Site for Testing Scoring Functions in Molecular Docking. J. Mol. Biol. 2002, 322, 339–355. 10.1016/S0022-2836(02)00777-5. 12217695

[ref33] Gu S.; Smith M. S.; Yang Y.; Irwin J. J.; Shoichet B. K. Ligand Strain Energy in Large Library Docking. J. Chem. Inf. Model. 2021, 61, 4331–4341. 10.1021/acs.jcim.1c00368. 34467754 PMC8501943

