# Supplementary material for: ZINC-22—A Free Multi-Billion-Scale Database of Tangible Compounds for Ligand Discovery
Source: J Chem Inf Model. 2023 Feb 15;63(4):1166–76. doi: 10.1021/acs.jcim.2c01253 (PMC9976280; doi:10.1021/acs.jcim.2c01253)
Supplement: Supplementary file 1 — ci2c01253_si_001.pdf [file ci2c01253_si_001.pdf]

# Supporting Information for

# ZINC-22 - A Free Multi-Billion-Scale Database of Tangible Compounds for Ligand Discovery

Benjamin I. Tingle+, Khanh G. Tang+, Mar Castanon+, John J Gutierrez, Munkhzul Khurelbaatar, Chinzorig Dandarchuluun, Yurii S. Moroz&, John J. Irwin*

Department of Pharmaceutical Chemistry, University of California San Francisco, 1700 4th *St, Mailcode 2550, San Francisco CA 94158-2330*

& Taras Shevchenko National University of Kyïv, 60 Volodymyrska Street, Kyïv 01601, UkraineNational Taras Shevchenko University of Kyiv, Kyiv 01601, Ukraine and Chemspace LLC (www.chem-space.com), 85 Chervonotkatska Street, Kyïv 02094, Ukraine.

+ these authors contributed equally

*Corresponding author jji@cgl.ucsf.edu

**Supporting Information Table of Contents**

S0. Access to databases to prevent molecules becoming unpatentable

S1. Source catalog contributions to ZINC-22

S2. Sharding script

S3. ZINC-22 numbering

S4. Software and Hardware overview

S5. Sn system overview

S6. Sb system overview

S7. Common Database Schema Overview

S8. Important management scripts for ZINC-22

S9. Files sizes in ZINC-22

S0. Access to databases to prevent molecules becoming unpatentable

To access the private databases, in addition to a login, a user id and password are required.
The user id is the acronym for G protein coupled receptors, all small case, four letter.
The password is a widely used four letter abbreviation of crystal, all small case, four letters.
We hope this helps prevent never-been-made molecules in these from losing their may-be-patented-for-composition-of-matter status.

**S1. Source catalog contributions to ZINC-22.** Number of SMILES in each, not accounting for duplication, and before stereochemical expansion.

| | S-Enamine | M-Enamine | WuXi | Mcule |
|---|---|---|---|---|
| H04 | 0 | 3 | 0 | 3 |
| H05 | 0 | 16 | 1 | 13 |
| H06 | 4 | 97 | 3 | 57 |
| H07 | 37 | 423 | 19 | 211 |
| H08 | 281 | 1,771 | 47 | 647 |
| H09 | 1,408 | 6,711 | 241 | 1,593 |
| H10 | 5,479 | 23,284 | 1,134 | 3,786 |
| H11 | 20,046 | 75,242 | 5,063 | 8,357 |
| H12 | 69,500 | 222,832 | 19,356 | 17,359 |
| H13 | 226,068 | 589,879 | 59,441 | 33,757 |
| H14 | 668,339 | 1,397,669 | 150,566 | 61,906 |
| H15 | 1,757,298 | 2,949,191 | 328,930 | 108,996 |
| H16 | 4,088,958 | 5,602,072 | 630,503 | 182,597 |
| H17 | 8,434,321 | 9,728,563 | 1,071,982 | 291,599 |
| H18 | 15,495,328 | 15,882,147 | 1,640,345 | 443,659 |
| H19 | 25,659,376 | 25,337,835 | 2,309,365 | 644,836 |
| H20 | 38,702,762 | 41,432,156 | 3,106,513 | 896,127 |
| H21 | 53,623,614 | 72,302,131 | 4,187,920 | 1,189,032 |
| H22 | 68,807,514 | 135,143,541 | 5,844,203 | 1,527,539 |
| H23 | 82,206,044 | 262,229,709 | 8,603,347 | 1,903,392 |
| H24 | 92,550,418 | 507,018,263 | 13,132,882 | 2,323,072 |
| H25 | 99,416,168 | 948,387,644 | 20,170,920 | 2,795,200 |
| H26 | 99,496,808 | 1,667,424,311 | 30,309,662 | 3,343,654 |
| H27 | 97,103,418 | 2,645,491,632 | 43,768,982 | 3,981,546 |
| H28 | 89,499,857 | 3,890,805,978 | 60,180,044 | 4,720,688 |
| H29 | 78,854,473 | 5,572,210,412 | 78,484,029 | 5,558,469 |
| H30 | 62,398,096 | 6,931,706,953 | 96,891,592 | 6,463,251 |
| | | | | |
| Sum ≤ H25 | 491,732,963 | 2,028,331,179 | 61,262,781 | 12,433,738 |
| Sum ≤ H30 | 919,085,615 | 22,735,970,465 | 370,897,090 | 36,501,346 |

## S2. Sharding script

https://github.com/docking-org/ZINC21-Tools/blob/master/rdkit_hlogp_batch_mp_2.py

```
import multiprocessing as mp
import sys
import os
import shutil
from rdkit.Chem import MolFromSmiles
from rdkit.Chem.Descriptors import MolLogP
from rdkit.Chem.SaltRemover import SaltRemover
from tqdm import tqdm

remover = SaltRemover()


def scale_logp_value(logp):
    if logp < -9.0:
        logp = -9.0
    elif logp > 9.0:
        logp = 9.0
    if logp < 0.0 or logp >= 5.0:
        logp = 100*int(logp)
    else:
        logp = 10*int(10*logp)
    return logp


def worker(line):
    smiles, cid = line.decode().strip().split()[:2]
    mol = MolFromSmiles(smiles)
    if mol:
        if '.' in smiles:
            mol = remover.StripMol(mol)
        logp = MolLogP(mol)
        num_heavy_atoms = mol.GetNumHeavyAtoms()
        if num_heavy_atoms > 99:
            num_heavy_atoms = 99
        sign = 'M' if logp < 0.0 else 'P'
        return f'{smiles} {cid}\n', len(line), True,
f'H{num_heavy_atoms:02}{sign}{abs(scale_logp_value(logp)):03}.txt'
    else:
        return f'{smiles} {cid}\n', len(line), False


if __name__ == '__main__':
    if len(sys.argv) != 2:
        print('Usage: python rdkit_hlogp_batch_mp_2.py <smiles>')
        exit()
    folder = os.path.dirname(sys.argv[1])
    name, ext = os.path.splitext(os.path.basename(sys.argv[1]))
```

```
    file_failed = os.path.join(folder, f'{name}_failed{ext}')
    tranches_folder = f'./{name}_tranches/'
    if os.path.exists(tranches_folder):
        shutil.rmtree(tranches_folder)
    os.makedirs(tranches_folder)
    cache = dict()
    cache_size_in_lines = 1024
    with open(sys.argv[1], 'rb') as f, open(file_failed, 'w',
newline='\n') as f_f, mp.Pool() as pool, \
            tqdm(total=os.path.getsize(sys.argv[1]), unit_scale=True,
unit_divisor=1024,
                 unit='B', mininterval=1.0) as _tqdm:
        for res in pool.imap(worker, f, chunksize=256):
            _tqdm.update(res[1])
            if res[2]:
                cache.setdefault(res[3], list()).append(res[0])
                if len(cache[res[3]]) >= cache_size_in_lines:
                    with open(os.path.join(tranches_folder, res[3]),
'a', newline='\n') as fout:
                        fout.writelines(cache[res[3]])
                    cache[res[3]].clear()
            else:
                f_f.write(res[0])
    for file_name, cached_lines in cache.items():
        if cached_lines:
            with open(os.path.join(tranches_folder, file_name), 'a',
newline='\n') as fout:
                fout.writelines(cached_lines)
    print('Done')
```

**S3. ZINC 3D numbering**

https://wiki.docking.org/index.php/ZINC22:Numbering

**S4. ZINC-22 Database Software and Hardware Overview**

Software Used**:** Python 3.6 (vanilla), Postgres 12.2, Bash

Hardware Overview

| Hostname | CPUS | Memory (B) | Subsystem | Database Instances |
|---|---|---|---|---|
| n-1-16 | 80 | 394652788 | Tin | 11 |
| n-1-17 | 80 | 394652836 | Tin | 10 |
| n-1-18 | 80 | 394652788 | Tin | 11 |
| n-1-19 | 80 | 196484320 | Tin | 10 |
| n-1-20 | 80 | 196484272 | Tin | 13 |
| n-1-21 | 80 | 790989768 | Tin | 13 |
| n-5-34 | 80 | 196484368 | Tin | 11 |
| n-5-35 | 80 | 394652832 | Tin | 10 |
| n-9-19 | 80 | 196499152 | Tin | 11 |
| n-9-20 | 80 | 196499152 | Tin | 10 |
| n-9-38 | 80 | 97415208 | Antimony | 16 |
| n-5-13 | 80 | 196499440 | Antimony | 16 |
| n-5-15 | 80 | 196499440 | Antimony | 16 |
| n-5-14 | 80 | 196499440 | Antimony | 16 |

### Subsystem Notes

The Tin subsystem contains the actual chemical data for zinc22, broadly partitioned by chemical properties. The antimony subsystem performs the auxiliary function of accelerating vendor code lookup on zinc22, as vendor codes do not typically contain any usable information about the chemicals they reference, so we must build up an additional system to map codes to the broad chemical partition(s) they are within.

**S5. Tin Database Schema Overview**

| Table Name | Partitioned | Partition Type | Description |
|---|---|---|---|
| substance | Yes | Hash (smiles) | Contains molecule SMILES data + substance ID information |
| catalog_content | Yes | Hash (supplier_code) | Contains vendor supplier code data + maps codes to vendors |
| catalog_substance | Yes | Hash (sub_id_fk) | Forms the relation between substance and catalog_content |
| catalog_substance_cat | Yes | Hash (cat_content_fk) | Same as above, but hashed by vendor ID for flexible lookup |
| substance_id | Yes | Hash (sub_id) | Maps substance ID to the substance partition smiles resides in. More info below. |
| catalog_id | Yes | Hash (cat_content_id) | Maps vendor ID to the catalog_content partition code resides in. |
| catalog | No | N/A | Contains more detailed information about vendors |
| meta | No | N/A | Contains details about TIN instance, e.g patch/upload history, # of partitions, etc. |
| transaction_record_{identifier} | No | N/A | Contains details about a particular upload transaction, one table for each transaction |

Note on the substance_id and catalog_id tables

Partitioning our primary tables by their unique key significantly speeds up bulk upload operations by allowing us to break up the task into manageable chunks. Unfortunately, this comes at the cost of slowing down most other query types, due to how Postgres handles partitions.

The problem is as follows- if a user is looking up (for example) a singular ZINC ID, Postgres needs to query each substance partition for that ID, thus potentially loading and checking hundreds of indexes for a singular lookup. This can be fixed by creating a secondary table that maps substance IDs to the partition they reside in, substance_id. This improves the performance of small queries by reducing the number of tables that are required to check in the worst case to two per lookup (substance_id partition & substance partition).
Similarly, catalog_id accelerates small lookups on the catalog_content table.

**S6. Antimony Database Schema Overview**

| Table Name | Partitioned | Partition Type | Description |
|---|---|---|---|
| supplier_codes | Yes | Hash (supplier_code) | Contains vendor code data + hash bucket |
| supplier_map | Yes | Hash (sup_id_fk, machine_id_fk) | Forms the relation between vendor code ID and machine ID |
| meta | No | N/A | Contains details about Antimony instance, e.g patch/upload history, # of partitions, etc. |
| transaction_record_{identifier} | No | N/A | Contains details about a particular upload transaction, one table for each transaction |

**S7. Common Database Schema Overview**

| Table Name | Description |
|---|---|
| antimony_hash_partitions | Maps SHA256 hash digits to logical antimony partition (last digits of SHA256 hash are used to sort vendor codes into Antimony database instances) |
| antimony_machines | Contains host/port details of each antimony instance & the logical partition said instance maps to |
| holdings_3d | Contains a list of all 3D tarballs produced for ZINC22, must be updated periodically. |
| tin_machines | Contains host/port details of each tin instance & canonical machine_id for said instance (used by Antimony) |
| tin_partitions | Contains the HAC+LogP start & end for Tin partitions. Tin is unique in that it is partitioned (on an instance level) by the chemical properties of substances contained within. |
| tranche_mappings | Maps each HAC+LogP tranche to a Tin instance |

Side Note: the common database is located @ zinc22user@n-1-17:5534:zinc22

**S8. Important Management Scripts**

These scripts for managing ZINC-22 in 2D are part of github.org/docking-org/zinc-22-2d

preprocessing/pre_process_all.bash

  - Initiates preprocessing of vendor chemical data. All data must be preprocessed before entering the zinc22 system

- utils-2d/tin/2d_export_all.bash
  - Exports database contents to disk, can choose one of the following three modes:
    - Substance export
      - Exports just SMILES and ZINC IDs to disk
    - Vendor export
      - Exports SMILES + Vendor Codes + ZINC IDs to disk
    - Antimony export
      - Exports Vendor Codes + machine_id to disk for future upload to antimony
- utils-2d/tin/2d_upload_all.bash
  - Initiates upload of tranched chemical data to Tin database instances. Must provide a unique identifier for uploaded data.
  - Uploads also produce a diff to a specified location in the event that rollbacks are desired.
- utils-2d/antimony/submit_all_sb_upload.sh
  - Initiates upload of exported antimony data to Antimony database instances.
- utils-2d/zinc22_stats/get_zinc22_upload_status.bash
  - Gets the latest successful upload for each tin database
- utils-2d/zinc22_stats/get_zinc22_patch_status.bash
  - Gets the pass/fail status for each patch on tin databases
- utils-2d/common_files
  - Okay, this one isn't a script, but it is referenced everywhere.
  - Contains all sorts of files that encapsulate the static configuration for the zinc22 system
  - Trying to move the contents of these files to the common database, but for now these files are still accurate and their contents are referenced by many scripts scattered around the zinc22 repository

**S9. Files sizes in ZINC-22**

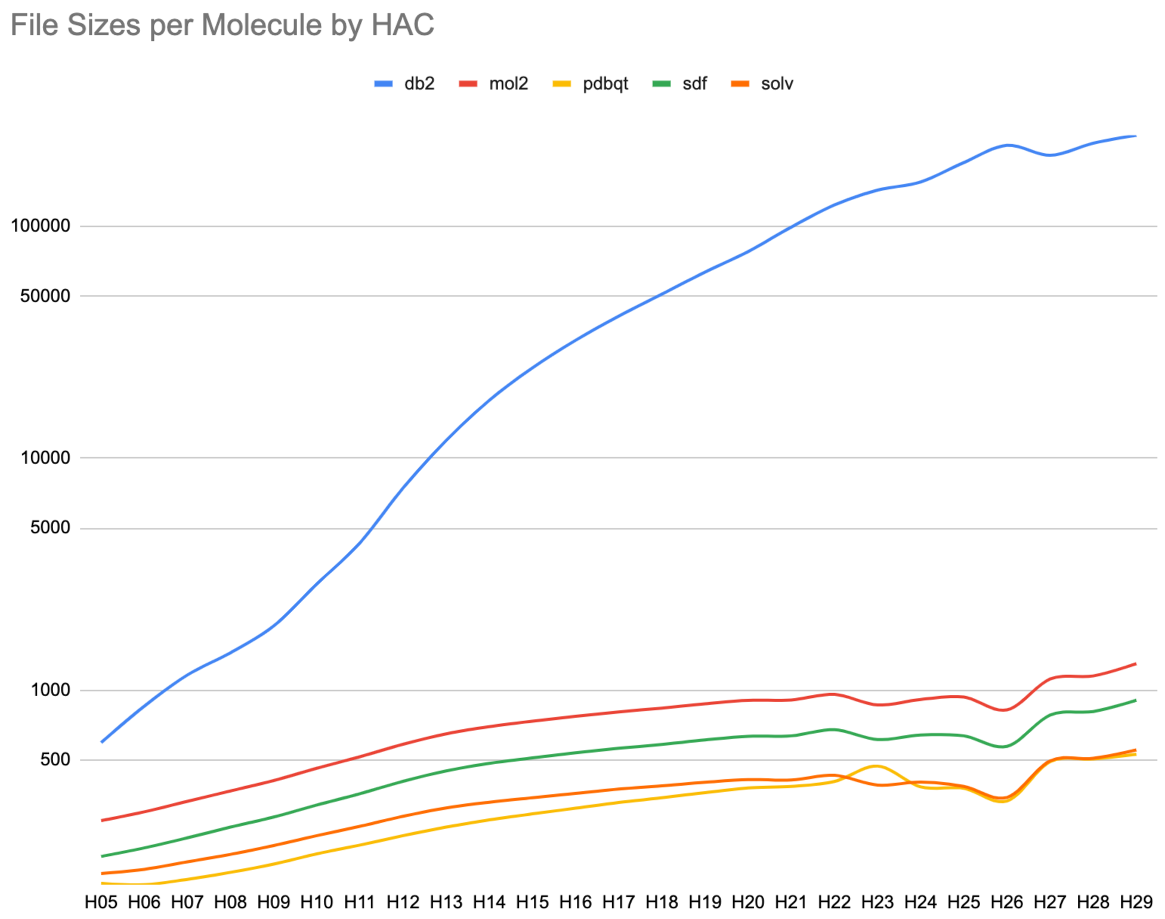


**Figure 9A. Average file size per molecule (Bytes), organized by heavy atom count. All files are gzipped.**

**Thus DB2 files show a sharp dependency on heavy atom count, due to the conformational sampling built into these files. The other file types show very slight sensitivity to heavy atom count.**

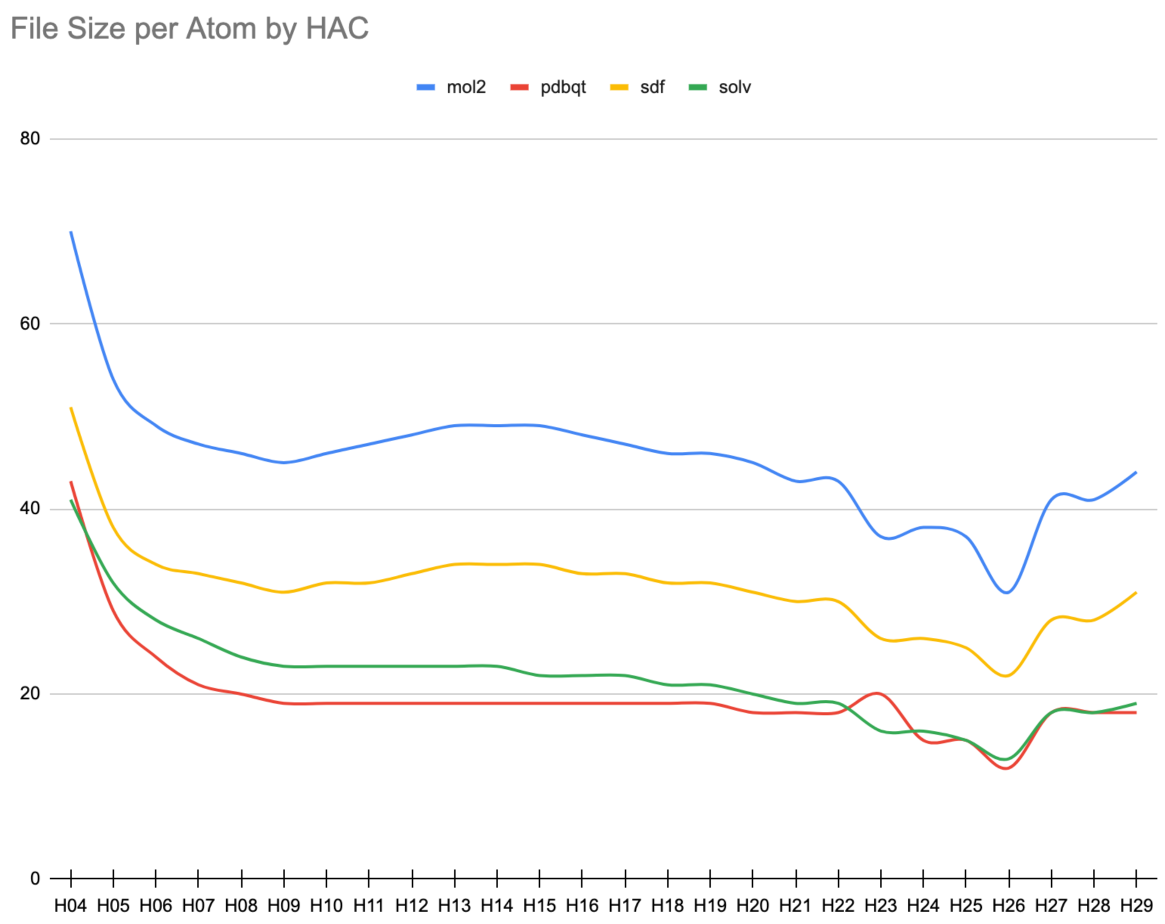


**Figure S9B. Number of bytes per atom, organized by heavy atom count. Files are gzipped. DB2 files are in figure S9C. Thus mol2 files are bigger than other file types, but by less than a factor of 2.**

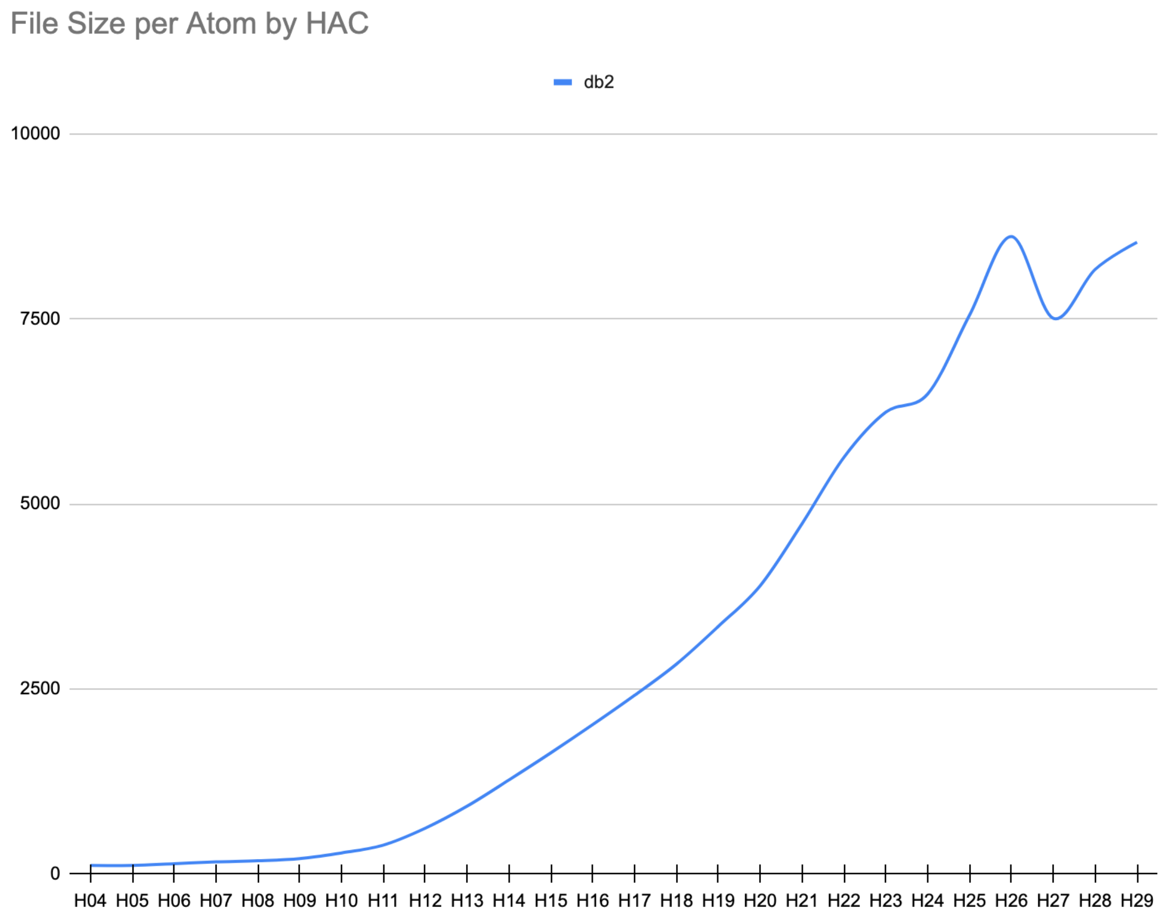


**Figure 9C. File size per atom, organized by heavy atom count. DB2 only. All files gzipped. For other file types see figure S9B**
